# Key Modulatory Role of Presynaptic Adenosine A_2A_ Receptors in Cortical Neurotransmission to the Striatal Direct Pathway

**DOI:** 10.1100/tsw.2009.143

**Published:** 2009-11-18

**Authors:** César Quiroz, Rafael Luján, Motokazu Uchigashima, Ana Patrícia Simoes, Talia N. Lerner, Janusz Borycz, Anil Kachroo, Paula M. Canas, Marco Orru, Michael A. Schwarzschild, Diane L. Rosin, Anatol C. Kreitzer, Rodrigo A. Cunha, Masahiko Watanabe, Sergi Ferré

**Affiliations:** ^1^ National Institute on Drug Abuse, IR , NIH, DHHS, Baltimore, MD, USA; ^2^ Departamento de Ciencias Médicas, Facultad de Medicina, Universidad de Castilla-La Mancha, Albacete, Spain; ^3^ Department of Anatomy, Hokkaido University School of Medicine, Sapporo, Japan; ^4^ Institute of Biochemistry, Faculty of Medicine, University of Coimbra, Coimbra, Portugal; ^5^ Gladstone Institute of Neurological Disease, Department of Physiology, University of California, San Francisco, USA; ^6^ Department of Neurology, MassGeneral Institute for Neurodegenerative Disease, Charlestown, USA; ^7^ Department of Pharmacology, University of Virginia Health Sciences Center, Charlottesville, USA

**Keywords:** adenosine A_2A_ receptor, striatum, basal ganglia, medium spiny neuron, glutamatergic neurotransmission, presynaptic receptors

## Abstract

Basal ganglia processing results from a balanced activation of direct and indirect striatal efferent pathways, which are controlled by dopamine D_1_ and D_2_ receptors, respectively. Adenosine A_2A_ receptors are considered novel antiparkinsonian targets, based on their selective postsynaptic localization in the indirect pathway, where they modulate D_2_ receptor function. The present study provides evidence for the existence of an additional, functionally significant, segregation of A_2A_ receptors at the presynaptic level. Using integrated anatomical, electrophysiological, and biochemical approaches, we demonstrate that presynaptic A_2A_ receptors are preferentially localized in cortical glutamatergic terminals that contact striatal neurons of the direct pathway, where they exert a selective modulation of corticostriatal neurotransmission. Presynaptic striatal A_2A_ receptors could provide a new target for the treatment of neuropsychiatric disorders.

### INTRODUCTION

The γ-aminobutyric-acidergic (GABAergic) striatal efferent neurons, also known as medium-sized spiny neurons (MSNs), constitute more than 95% of the striatal neuronal population[1]. According to their selective peptide expression, MSNs are subdivided into enkephalinergic and substance P-dynorphinergic neurons, which give rise to two striatal efferent systems that connect the striatum with the output structures of the basal ganglia: the substantia nigra pars reticulata and the internal segment of the globus pallidus (entopeduncular nucleus in rodents)[1]. These are called “direct” and “indirect” efferent pathways. The direct pathway consists of substance P-dynorphinergic MSNs, which directly connect the striatum with the output structures. The indirect pathway originates with enkephalinergic MSNs, which connect the striatum with the external segment of the globus pallidus (globus pallidus in rodents). GABAergic neurons of the external segment of the globus pallidus provide inputs to the output structures of the basal ganglia and to the subthalamic nucleus, which also connects with the output structures[1,2]. It is generally accepted that smooth motor drive results from the counterbalanced influence of the direct and indirect pathways on the neuronal activity in the output structures, with stimulation of the direct pathway resulting in motor activation and stimulation of the indirect pathway resulting in motor inhibition[2,3,4].

It is now widely accepted that striatal dopamine $D_1$ and $D_2$ receptors are largely segregated and predominantly expressed in direct- and indirect-pathway MSNs, respectively[1,5,6]. Dopamine induces motor activation by simultaneously activating the direct pathway (acting on stimulatory $D_1$ receptors localized in direct-pathway MSNs) and depressing the indirect pathway (acting on inhibitory $D_2$ receptors localized in indirect-pathway MSNs)[5,6,7,8,9]. In addition to dopamine, adenosine is an important modulator of the function of striatal GABAergic efferent neurons. Adenosine $A_{2A}$ receptors are more densely expressed in the striatum than anywhere else in the brain[10] and striatal postsynaptic $A_{2A}$ receptors are selectively localized in the dendritic membranes of the indirect-pathway MSNs, where they can heteromerize and establish strong functional antagonistic interactions with $D_2$ receptors[9,11,12,13,14]. Stimulation or blockade of striatal postsynaptic $A_{2A}$ receptors counteracts and potentiates, respectively, the effects induced by $D_2$ receptor activation in the indirect-pathway MSN[7,11,14]. At the behavioral level, stimulation or blockade of striatal postsynaptic $A_{2A}$ receptors counteracts and potentiates, respectively, the motor activating effects of $D_2$ receptor stimulation (reviewed in [15]).

Striatal presynaptic adenosine $A_{2A}$ receptors have also been identified in glutamatergic terminals, where they heteromerize with $A_1$ receptors and perform a fine-tuned modulation of glutamate release[16]. However, it was assumed that these presynaptic $A_{2A}$ receptors were localized in most striatal glutamatergic terminals, without any preference for those contacting direct- or indirect-pathway MSNs. The present study demonstrates that presynaptic $A_{2A}$ receptors are preferentially localized in cortical glutamatergic terminals that contact direct-pathway MSNs and that they are particularly involved in a selective modulation of cortical neurotransmission to the direct striatal pathway.

### MATERIALS AND METHODS

#### Animals

$A_{2A}$ receptor knockout mice and their wild-type littermates with a congenic C57BL/6 background (2–4 months old) were used for the immunohistochemical studies in striatal sections. M4-GFP and D2-GFP heterozygotic bacterial artificial chromosome (BAC) transgenic mice (20–30 days old) were used for the electrophysiological experiments in brain slices. The generation and characterization of the transgenic mice have been described previously[17,18]. Male Wistar rats (6–8 weeks old) were used in the experiments with striatal synaptosomes and purified nerve terminals. Male Sprague-Dawley rats (8–12 weeks old) were used in the experiments with cortical electrical stimulation and in the immunohistochemical experiments for colocalization of $A_{2A}$ receptors and vGluT1 (vesicular glutamate transporter type 1). All animal experiments were performed according to the ethical guidelines of the National Institutes of Health, European Union, and Hokkaido University, Japan. Although different rodent species, strains, and ages were used, which were optimal for the different experimental procedures, it is very improbable that they could distort the congruent final qualitative finding, i.e., a clear predominant presynaptic $A_{2A}$ receptor modulation of cortical neurotransmission to the direct striatal pathway.

### Immunohistochemistry of Striatal Sections

Mice were fixed transcardially under deep pentobarbital anesthesia (100 mg/kg body weight, i.p.) with 4% formaldehyde in 0.1 *M* sodium phosphate buffer (PB), pH 7.2, for immunofluorescence; with 4% formaldehyde/0.1% glutaraldehyde in PB for single-labeling electron microscopy; and with 4% formaldehyde + 0.05% glutaraldehyde + 15% (v/v) saturated picric acid made up in 0.1 *M* PB, pH 7.4, for double-labeling electron microscopy. The following affinity-purified primary antibodies were used: antimouse $A_{2A}$ receptor (raised in rabbit, guinea pig, and goat; characterized in the present study), antimouse $D_1$ receptor (raised in guinea pig and goat[19]), antimouse $D_2$ receptor (raised in rabbit and guinea pig[19]), and antimouse microtubule-associated protein-2 (MAP2, raised in goat[19]). Antibodies against $A_{2A}$ receptors were produced against the C-terminal 30-amino-acid sequence (GenBank, accession number, NM009630), fused to glutathione *S*-transferase (GST), and affinity-purified using GST-free peptides coupled to CNBr-activated Sepharose 4B (Amersham Biosciences; Piscataway, NJ). Commercial antibodies against substance P (Chemicon International, Temucula, CA) and Leu-enkephalin (Chemicon) were also used. For light microscopy immunofluorescence experiments, 50-μm-thick microslicer sections were obtained with a Leica VT1000S microtome (Nussloch, Germany) and incubated with 10% normal donkey serum for 20 min, a mixture of primary antibodies overnight (1 μg/ml), and a mixture of Alexa Fluor-488-, indocarbocyanine (Cy3)-, and indodicarbocyanine (Cy5)-labeled species-specific secondary antibodies for 2 h at a dilution of 1:200 (Invitrogen, Grand Island, NY; Jackson ImmunoResearch, West Grove, PA). Phosphate-buffered saline (PBS) containing 0.1% Tween 20 was used to dilute antibodies and as washing buffer. Images were taken with a fluorescence microscope (AX-70; Olympus Optical, Tokyo, Japan) equipped with a digital camera (DP70; Olympus Optical) or with a confocal laser scanning microscope (FV1000; Olympus Optical). For pre-embedding, single- and double-labeling electron microscopy 50- to 60-μm-thick microslicer sections were obtained (Leica VT1000S), respectively. In single-labeling electron microscopy experiments, microslicer sections were dipped in 5% bovine serum albumin (BSA)/0.02% saponin/Tris buffered saline (TBS) for 30 min, and incubated overnight with guinea pig anti–$A_{2A}$ receptor antibody (1 μg/ml) diluted with 1% BSA/0.004% saponin/TBS and then with anti–guinea pig IgG linked to 1.4-nm gold particles (Nanogold; Nanoprobes, Stony Brook, NY) for 2 h. Immunogold was intensified with a silver-enhancement kit (HQ silver; Nanoprobes), treated with 1% osmium tetroxide for 15 min, stained in block with 2% uranyl acetate for 20 min, dehydrated, and embedded in Epon 812. Photographs were taken with an H-7100 electron microscope (Hitachi, Tokyo, Japan). The number of immunogold particles for $A_{2A}$ receptors were counted in profiles of dendrites, spines, and terminals using an IPLab software (Nippon Roper, Tokyo, Japan). For double-labeling, pre-embedding, electron microscopy experiments, free-floating sections were incubated in 10% normal goat serum (NGS) diluted in TBS for 1 h. Sections were then incubated for 48 h in a mixture of two antibodies ($A_{2A}$ and $D_1$ or $A_{2A}$ and $D_2$ receptors), at a final protein concentration of 1–2 μg/ml each, diluted in TBS containing 1% NGS. Then, one of the primary antibodies (anti–$D_1$- or anti–$D_2$-receptor antibodies) was visualized by the immunoperoxidase reaction and the other (anti–$A_{2A}$ receptor antibody) by the silver-intensified immunogold reaction. After primary antibody incubation, the sections were incubated at 4°C overnight in a mixture of the following secondary antibodies: goat antirabbit (Fab fragment, diluted 1:100) coupled to 1.4 nm gold (Nanoprobes), goat anti–guinea pig (Fab fragment, diluted 1:100) coupled to 1.4 nm gold (Nanoprobes), biotinylated goat antirabbit (diluted 1:100; Vector Laboratories, Burlingame, CA), and biotinylated goat anti–guinea pig (diluted 1:100; Vector Laboratories) antibodies, all of them made up in TBS containing 1% NGS. After washes in TBS, sections were washed in double-distilled water, followed by silver enhancement of the gold particles with an HQ Silver kit (Nanoprobes) for 8–10 min. Subsequently, the sections were incubated for 4 h in the avidin-biotin complex (Vector Laboratories) made up in TBS and then washed in Tris buffer (TB). Peroxidase was visualized with diaminobenzidine (0.05% in TB, pH 7.4) using 0.01% $H_2O_2$ as substrate for 5–10 min. The sections were washed in PB and then postfixed with $OsO_4$ (1% in 0.1 *M* PB), followed by block staining with uranyl acetate, dehydration in graded series of ethanol, and flat-embedding on glass slides in Durcupan resin

(Fluka, Buchs, Switzerland) and the ultrastructural analysis was performed in a Jeol-1010 electron microscope (Jeol, Tokyo, Japan). The methods for the experiments with dual immunoperoxidase and immunogold labeling for $A_{2A}$ receptor (with the previously characterized monoclonal anti–$A_{2A}$ receptor antibody directed against full-length purified receptor[20]; 2.5 μg/ml) and v-GluT1 (rabbit anti-vGluT1; a gift from Robert H. Edwards, UCSF, San Francisco, CA; 1:3000) in striatal sections form Sprague-Dawley rats are described in detail elsewhere[21,22].

### Immunocytochemistry of Striatal Synaptosomes and Purified Nerve Terminals

Rats were anesthesized under halothane atmosphere before being killed by decapitation. Brains were removed and the striata dissected out. Synaptosomes were obtained through a sequential centrifugation using sucrose and Percoll media, as previously described[23]. Briefly, striatal tissue was homogenized at 4°C in sucrose solution (0.32 *M*) containing 50 m*M* Tris-HCl, 2 m*M* ethylene glycol tetraacetic acid (EGTA), and 1 m*M* dithiothreitol, pH 7.6. The resulting homogenate was centrifuged at 3000 x *g* for 10 min at 4°C, the supernatant collected and centrifuged at 14,000 x *g* for 20 min at 4°C. The pellet was resuspended in 1 ml of a 45% (v/v) Percoll solution made up in a Krebs solution (composition 140 m*M* NaCl, 5 m*M* KCl, 25 m*M* HEPES, 1 m*M* ethylenediaminetetraacetic acid (EDTA), 10 m*M* glucose, pH 7.4). After centrifugation at 14,000 x *g* for 2 min at 4°C, the top layer was removed (synaptosomal fraction) and washed in 1 ml Krebs solution. Purified nerve terminals were obtained through a discontinuous Percoll gradient (modified from methods in [24]). Briefly, striatal tissue was homogenized in a medium containing 0.25 *M* sucrose and 5 m*M* Tris/EDTA/saline (TES) (pH 7.4). The homogenate was spun for 3 min, 2000 x *g,* at 4ºC and the supernatant spun again at 9500 x *g* for 13 min. Then, the pellets were resuspended in 8 ml of 0.25 *M* sucrose and 5 m*M* TES (pH 7.4), and 2 ml were placed onto 3 ml of Percoll discontinuous gradients containing 0.32 *M* sucrose, 1 m*M* EDTA, 0.25 m*M* dithiothreitol, and 3, 10, or 23% Percoll, pH 7.4. The gradients were centrifuged at 25,000 x *g* for 11 min at 4ºC. Nerve terminals were collected between the 10 and 23% Percoll bands, and diluted in 15 ml of HEPES-buffered medium (140 m*M* NaCl, 5 m*M* KCl, 5 m*M* $NaHCO_3$, 1.2 m*M* $NaH_2PO_4$, 1 m*M* $MgCl_2$, 10 m*M* glucose, and 10 m*M* HEPES, pH 7.4). After centrifugation at 22,000 x *g* for 11 min at 4ºC, the nerve terminal pellet was removed. This procedure for preparation of the purified nerve terminals (in the absence of calcium) is crucial to allow reducing the amount of postsynaptic density material[24]. Striatal synaptosomes and purified nerve terminals were placed onto coverslips previously coated with poly L-lysine, fixed with 4% formaldehyde for 15 min, and washed twice with PBS medium (140 m*M* NaCl, 3 m*M* KCl, 20 m*M* $NaH_2PO_4$, 15 m*M* $KH_2PO_4$, pH 7.4). These preparations were then permeabilized in PBS with 0.2% Triton X-100 for 10 min and then blocked for 1 h in PBS with 3% BSA and 5% normal rat serum. Both preparations were then washed twice with PBS and incubated with different combinations of the following primary antibodies for 1 h at 23–25ºC: goat anti–$A_{2A}$ receptor antibody (1:300, from Santa Cruz Biotechnology-Europe, Freelab, Lisbon, Portugal), mouse anti-PSD-95 (1:300, from Chemicon, Millipore-Portugal, Lisbon), mouse antisynaptophysin (1:200, from Sigma, Sintra, Portugal), rabbit antisynaptophysin (1:200 from Chemicon), mouse anti-SNAP-25 (1:300, from Sigma), mouse anti-GFAP (1:500 from Sigma), mouse anti-vGluT1 (1:1000 from Synaptic Systems, Goettingen, Germany), and the anti–$D_1$ receptor antibody (1:500) and anti–$D_2$ receptor antibody (1:500) described above[19]. The synaptosomes and purified nerve terminals were then washed three times with PBS with 3% BSA and incubated for 1 h at room temperature with AlexaFluor-488 (green)– or AlexaFluor-594 (red)–labeled donkey antimouse IgG antibody (1:200), AlexaFluor-594–labeled goat anti–guinea pig IgG (1:200), AlexaFluor-594–labeled donkey antirabbit IgG (1:200), and AlexaFluor-350 (blue)–labeled donkey antigoat IgG antibodies (1:50); all from Invitrogen (Eugene, OR). After washing and mounting onto slides with Prolong Antifade, the preparations were visualized in a Zeiss Axiovert 200 inverted fluorescence microscope equipped with a cooled, charge-coupled device (CCD) camera (Zeiss, Gottingen, Germany) and analyzed with MetaFluor 4.0 software (Molecular Devices, Union City, CA). Each coverslip was analyzed by counting three different fields and in each field, a total amount of 100 individualized elements.

### Electrophysiology in Brain Slices

Coronal slices (300-µm thick) containing the dorsal striatum were prepared from the brains of $D_2$-GFP and $M_4$-GFP BAC transgenic mice. Slices were superfused with artificial cerebrospinal fluid (ACSF) solution containing (in m*M*): 125 NaCl, 2.5 KCl (or 4.5 KCl where noted), 2 $CaCl_2$, 1 $MgCl_2$, 26 $NaHCO_3$, 1.25 $NaH_2PO_4$, and 12.5 glucose, bubbled with 95% $O_2$/5% $CO_2$. Picrotoxin (50 µ*M*) was added to the external solution to suppress synaptic currents mediated by $GABA_A$ receptors. Slices were stored at room temperature until recording. All recordings were performed at a temperature of 30–32°C. Whole-cell voltage-clamp recordings were obtained using infrared differential interference contrast and fluorescence microscopy. MSNs were identified by their fluorescence. Green fluorescence protein (GFP)–positive MSNs in the $M_4$-GFP BAC transgenic line were used to identify direct-pathway neurons. In a few cases, GFP-negative GABAergic efferent neurons in the $D_2$-GFP line were also considered as direct-pathway neurons. GFP-positive MSNs in the $D_2$-GFP BAC transgenic line were used to identify indirect-pathway neurons. Glass electrodes (2.5–3.5 MΩ) were filled with a solution containing (in m*M*): 120 $CsMeSO_3$, 15 CsCl, 8 NaCl, 0.2 EGTA, 10 HEPES, 2 Mg-ATP, 0.3 Na-GTP, 10 triethanolamine, 5 QX-314, adjusted to pH 7.3 with CsOH. Access resistance and holding current (current required to hold neurons at –70 mV) were monitored continuously and experiments were rejected if these parameters changed by more than 15% during recording. To stimulate glutamatergic afferents, small glass electrodes (tip diameter: 10–20 µm) were filled with external saline and placed between the recorded GABAergic efferent neuron and cortex, typically ~50–100 µm from the cell body. Stimulus intensities did not exceed 10–15 µA under these conditions. The stimulus isolator was triggered by computer-controlled transistor-transistor logic outputs. Voltage-clamp recordings were performed with a multiclamp 700B (Molecular Devices), filtered at 2 kHz and digitized at 10 kHz. Acquisition and analysis were performed using custom Igor Pro software (WaveMetrics, Lake Oswego, OR).

### Mean-Variance Analysis

Fluctuations in excitatory postsynaptic current (EPSC) amplitude can be expressed by the coefficient of variation, CV = SD/mean, which is independent of quantal size (*q*) according to models of synaptic transmission (*q*, mean postsynaptic response to a single vesicle of neurotransmitter; *p*, probability of release; *N*, number of release sites)[25,26]. The ratio of squared CVs before ($CV_{control}^2$) and after ($CV_{drug}^2$) drug application and the ratio of the mean EPSC amplitudes after ($EPSC_{drug}$) and before ($EPSC_{control}$) drug application were calculated. The relation of these two ratios indicates which synaptic parameter has changed during the experiment. Increases in mean EPSC amplitude yield the following possibilities: (1) ($CV_{control}^2/CV_{drug}^2$) > $EPSC_{drug}/EPSC_{control}$, which suggests an increase in *p* and/or *N*; (2) ($CV_{control}^2/CV_{drug}^2$) = $EPSC_{drug}/EPSP_{control}$, which suggests an increase in *N*; or (3) ($CV_{control}^2/CV_{drug}^2$) = 1, which suggests an increase in *q*. Decreases yield the following possibilities: (1) ($CV_{control}^2/CV_{drug}^2$) < $EPSC_{drug}/EPSC_{control}$, which suggests a decrease in *p* and/or *N;* (2) ($CV_{control}^2/CV_{drug}^2$) = $EPSC_{drug}/EPSP_{control}$, which suggests a decrease in *N*; or (3) ($CV_{control}^2/CV_{drug}^2$) = 1, which suggests a decrease in *q*.

### Surgical Procedures

Rats were anesthetized with 3 ml/kg of Equithesin (4.44 g of chloral hydrate, 0.972 g of Na pentobarbital, 2.124 g of $MgSO_4$, 44.4 ml of propylene glycol, 12 ml of ethanol and sterile $H_2O$ up to 100 ml of final solution; NIDA Pharmacy, Baltimore, MD) and implanted unilaterally with bipolar stainless-steel electrodes, 0.15 mm in diameter (Plastics One, Roanoke, VA), into the orofacial area of the lateral agranular motor cortex (3 mm anterior, 3 and 4 mm lateral, and 4.2 mm below bregma). The electrodes and a head holder (connected to a swivel during stimulation) were fixed on the skull with stainless-steel screws and dental acrylic resin. For the *in vivo* microdialysis experiments, concentric microdialysis probes (built as described in [27]) with 2-mm-long dialysis membranes were also implanted (during the same surgical procedure) in the ipsilateral striatum (with respect to bregma: 0 mm anterior, 2.8 or 4.5 mm lateral, and 7 mm below). For the experiments with electromyographic (EMG) recording, electrodes were also implanted in mastication muscles (during the same surgical procedure). Two 5-mm-long incisions were made in the skin on the upper and lower jaw areas to expose the masseter and the lateral pterygoid muscles. Two silicon rubber–coated coiled stainless-steel recording electrodes (Plastics One) were pushed under the skin from the incision in the skull up to the incisions in the jaw. The electrodes were implanted in the masseter and the lateral pterygoid muscles, and the skin was closed with surgical staples. The other end of the recording electrodes was encased in a molded plastic pedestal with a round threaded post attached to an electrical swivel and a differential amplifier (Grass LP511; Grass Instruments, West Warwick, RI). The pedestal was secured to the skull with dental cement, together with the stimulation electrodes.

### Cortical Electrical Stimulation

Five to six days after surgery, the animals were placed in individual bowl chambers and the implanted electrodes were attached to an electrical stimulator (Grass S88K; Grass Instruments). The parameters of cortical stimulation were the same as those shown previously to induce phosphorylation of ERK1/2 in cells of the projecting striatal area, the lateral caudate-putamen[28]. After 10 min of habituation, biphasic current pulse trains (pulse of 0.1 msec, 150–200 μA, 100 Hz, 160-msec trains repeating once per second) were delivered using two-coupled constant current units (Grass PSIU6; Grass Instruments). The intensity was 150 μA for most cases or it was increased (stepwise 5 μA increases in the intensity) up to 200 μA, until small jaw movements were observed. The animals that failed to show visible somatic movements (less than 10%) were excluded from additional analysis. In no case did animals display evidence of seizure activity from the electrical stimulation.

### *In Vivo* Microdialysis

The experiments were performed on freely moving rats 24 h after probe implantation. A Ringer solution of (in m*M*) 144 NaCl, 4.8 KCl, 1.7 $CaCl_2$, and 1.2 $MgCl_2$ was pumped through the dialysis probe at a constant rate of 1 μl/min. After a washout period of 90 min, dialysate samples were collected at 20-min intervals and split into two fractions of 10 μl, to measure glutamate and dopamine separately. After 60 min of collecting samples for baseline, the rats were perfused with the $A_{2A}$ receptor antagonist 3,7-dihydro-8-[(1E)-2-(3-methoxyphenyl)ethenyl]-7-methyl-3-[3-(phosphonooxy)propyl-1-(2-propynyl)-1H-purine-2,6-dione disodium salt hydrate (MSX-3; 1 μM) or the $D_1$ receptor antagonist R(+)-7-chloro-8-hydroxy-3-methyl-1-phenyl-2,3,4,5-tetrahydro-1H-3-benzazepine hydrochloride (SCH23390; 1 μ*M*) dissolved in the Ringer solution. After 2 h of drug perfusion and sample collection, electrical stimulation pulses were applied through the electrodes implanted in the orofacial motor cortex for 20 min and samples were collected for 2 additional hours. At the end of the experiment, rats were killed with an overdose of Equithesin and methylene blue was perfused through the probe. The brain was removed and placed in a 4% formaldehyde solution, and coronal 50-μm-thick sections were cut and stained with cresyl violet to verify probe and electrode location. Dopamine content was measured by high-pressure liquid chromatography (HPLC) coupled to an electrochemical detector, as described in detail previously[27]. Glutamate content was measured by reverse-phase HPLC coupled to a flourimetric detector as described previously[29]. The limit of detection (which represents three times baseline noise levels) for dopamine and glutamate was 0.5 and 50 n*M*, respectively. Dopamine and glutamate values were transformed as percentage of the mean of the three values before the stimulation and transformed values were statistically analyzed.

### EMG Recording and Power Correlation Analysis

Five days after surgery, rats were placed in individual bowl chambers. Both the stimulation electrodes and the recording electrodes were attached using flexible shielded cabling to a four-channel electrical swivel and then the stimulation electrodes were connected to two-coupled constant current isolation units (PSIU6; Grass Instruments) driven by an electrical stimulator (Grass S88K; Grass Instruments). The recording electrodes were connected to a differential amplifier (Grass LP511; Grass Instruments). This configuration allows the rat to move freely while the stimulation and EMG recordings are taking place. After 10 min of habituation, biphasic current pulse trains (pulse of 0.1 msec, 150–200 μA, 100 Hz, 160-msec trains repeating once per 2 sec) were delivered. The current intensity was adjusted to the threshold level, defined as the minimal level of current intensity allowing at least 95% of the stimulation pulses to elicit a positive EMG response. Positive EMG response was defined as at least a 100% increase of the peak-to-peak amplitude respective to the background tonic EMG activity lasting more than 100 msec, or at least a 70% increase in the power of the EMG signal respective to the baseline. Positive EMG responses always matched observable small jaw movements. The threshold level was different for each animal, but it was very stable and reproducible once established. The threshold level was in the 100–150 μA range for 90% of the animals and it reached 200 μA in a few (five) animals. Animals that failed to show a positive EMG response with electrical cortical stimulation intensities of 200 μA were discarded from the experimental procedure (less than 10%). Both stimulator monitoring, and the amplified and filtered EMG signal (20,000 times gain, bandwidth from 10 to 1,000 Hz with a notch filter set at 60 Hz), were directed to an analog digital converter (Lab-trax, WPI, Sarasota, FL) and digitized at a sampling rate of 10,000 samples/sec. Recordings of the digitized data were made with Data-trax software (WPI). A power correlation analysis was used to quantify the correlation between the stimulation pulses of current delivered into the orofacial motor cortex (input signal; μA) and the elicited EMG response in the jaw muscles (output signal; μV). Increases or decreases in the power correlation coefficient (PCC) between these two signals were meant to describe increases or decreases in the efficacy of the transmission in the neural circuit, respectively. Off line, both signals were rectified and the root mean square (RMS) over each period of the stimulation pulses was calculated in the recorded signals using Data-trax software (WPI). The transformed data (RMS) from the stimulator monitor and the EMG were then exported with a time resolution of 100 samples/sec to a spreadsheet file. The stimulation signal values were used as a reference to select data in a time window of 320 msec, starting at the beginning of each train of pulses. This time window was chosen to ensure the analysis of any EMG response whose occurrence or length was delayed from the onset of the stimulation trains, and to maximize the exclusion from the analysis of spontaneous jaw movements not associated with the stimulation. Pearson's correlation between the RMS values from the stimulation and EMG signals was then calculated for each experimental subject. The effect of the systemic administration (i.p.) of MSX-3 or SCH23390 (dissolved in saline) was studied in independent experiments. PCC was calculated using the data recorded 40 or 10 min after the administration of different doses of MSX-3 (and saline) or SCH23390 (and saline), respectively. The difference in the times of analysis corresponded to the initial time of action for each drug.

### Analysis of Striatal Protein Phosphorylation

Immediately after the offset of 20 min of cortical stimulation, the animals were decapitated and the brains were rapidly extracted, frozen in dry ice-cold isopentane, and stored at –80°C. Subsequently, unilateral tissue punches of the lateral striatum (16 gauge) at the anteroposterior level of bregma 0.0, corresponding to the area with maximal expression of phosphorylated ERK1/2[28], were obtained from ~1-mm-thick coronal sections cut in a cryostat at –20°C. The rostral side of the coronal sections was localized approximately between bregma 0.5 mm and bregma –0.5 mm. Tissue punches were processed to determine total and phosphorylated ERK1/2, and total and phosphorylated $GluR_1$ (at $Ser_{845}$), by Western blotting as described in detail elsewhere[29]. The band intensities for each of the test samples quantified were always within the range of the standard curve. For each animal, the values obtained from the experiments with phosphorylated and total ERK1/2 corresponded to the addition of the band intensities of both ERK1 and ERK2. For each animal, the values of phosphorylated ERK1/2 and phosphorylated $GluR_1$ were normalized (as percentage of control) to total ERK1/2 and total $GluR_1$ subunit of the AMPA receptor, respectively. In each Western blot, all values were normalized (as percentage of control) with respect to the standard curve.

### Statistical Analysis

In the immunohistochemical electron microscopy experiments, differences in the number of immunogold particles from the different profiles (dendrites, spines, and nerve terminals) in striatal sections from wild-type vs. $A_{2A}$ receptor knockout mice were analyzed by nonpaired Student's *t* test. In the immunocytochemical experiments, differences in the percentage of $D_1$ receptor–expressing striatal synaptosomes and purified nerve terminals were analyzed by nonpaired Student's *t* test. In the electrophysiological experiments in brain slices, the statistical significance for the effects of $A_{2A}$ receptor agonists and antagonists on EPSC amplitude was calculated using Student's paired *t* test. *In vivo* microdialysis-transformed data (percentage of the mean of the three values before the stimulation) were statistically analyzed with a two-way repeated measures ANOVA, with treatment factor (four levels, controls in lateral and medial striatum, and MSX-3 and SCH23390 in the lateral striatum) and time (two levels, before and after stimulation), followed by Newman-Keuls tests, to compare glutamate and dopamine values from the samples obtained just before and after cortical electrical stimulation between different groups. The effects of the $A_{2A}$ and $D_1$ receptor antagonists on PCC and striatal protein phosphorylation were analyzed by one-way ANOVA, followed by Newman-Keuls test.

## RESULTS

### Immunohistochemical Analysis of Striatal $A_{2A}$ Receptors

Western blot analysis showed a major predominant band around the expected 44 kDa (Fig. 1a). Single-immunofluorescence experiments demonstrated selective labeling of the dorsal and ventral striatal areas of wild-type mice, confirming the presence of the $A_{2A}$ receptor in these regions[20] (Fig. 1b) and its absence in their $A_{2A}$ receptor knockout littermates (Fig. 1c). Double- and triple-immunofluorescence experiments further validated the specificity of the $A_{2A}$ receptor antibody. In double-immunofluorescence experiments with the neuronal marker MAP2, $A_{2A}$ receptors were localized in some, but not all neurons with the characteristic size of the MSNs (Fig. 1d). Triple-immunofluorescence techniques showed $A_{2A}$ receptors colocalized with enkephalin, but not with substance P (Fig. 1e,f), as expected from previous *in situ* hybridization studies[30,31,32]. Also, we found a mutually exclusive pattern of $A_{2A}$ and $D_1$ receptor immunofluorescences (in red and blue, respectively; Fig. 1g) and, as previously reported[19], between $D_2$ and $D_1$ receptor immunofluorescences (in green and blue, respectively; Fig. 1g). On the other hand, there was a strong colocalization between $A_{2A}$ and $D_2$ receptors (in yellow; Fig. 1g).

Electron microscopy with silver-enhanced pre-embedding immunogold in mouse striatum showed a predominant localization of $A_{2A}$ receptors in the cell surface of some, but not all, somatodendritic elements (Fig. 2). Among those elements, the density of immunogold labeling was apparently higher in dendrites than in spines or somata (Fig. 2a,c,d,g). Low $A_{2A}$ receptor labeling was occasionally detected in terminals forming asymmetrical synapses with unlabeled and less commonly with labeled spines (Fig. 2e,f). In agreement with previous results using another $A_{2A}$ receptor antibody[21], the quantitative analysis showed that the density of immunogold particles (particles/$\mu m^2$) is higher in dendrites than in spines and higher in spines than in terminals (only terminals forming asymmetrical synapses were counted). In the previous study[21], $A_{2A}$ receptor immunoreactivity was achieved by using the avidin-biotin peroxidase method, which is more sensitive than the immunogold detection used in the present study. This

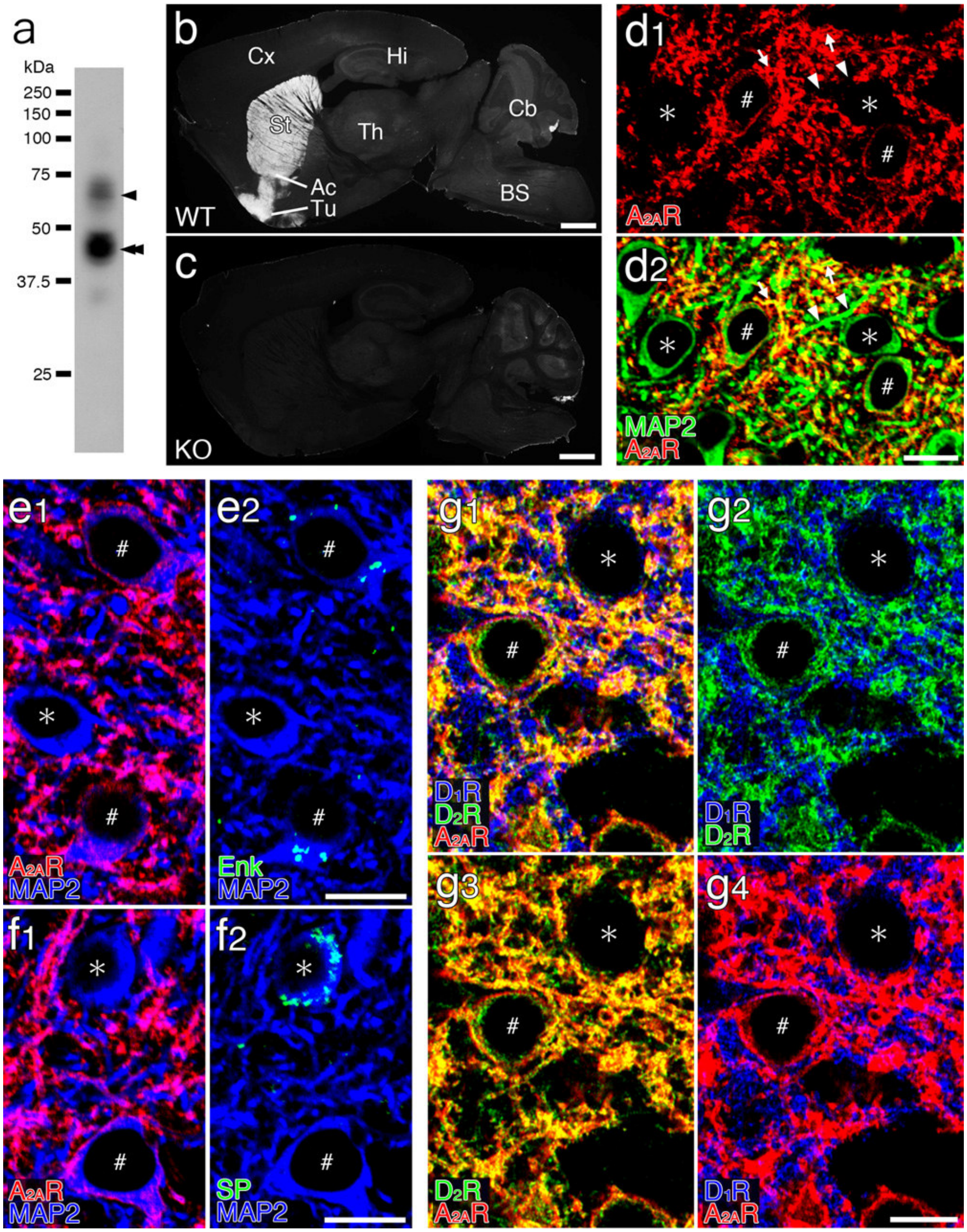

**FIGURE 1.** Characterization of anti–$A_{2A}$ receptor antibody. (a) Immunoblot analysis with striatal protein sample showing that anti–$A_{2A}$ receptor antibody recognizes major (double arrowhead) and minor (single arrowhead) protein bands at about 44 and 62 kDa, respectively. (b,c) Single immunofluorescence of parasagittal brain sections showing an exclusive immunoreactivity in the striatum (St), nucleus accumbens (Ac), and olfactory tubercule (Tu) of the wild-type mice (WT), but not $A_{2A}$ receptor knockout mice (KO); Cx, cortex; Hi, hippocampus; Th, thalamus; Cb, cerebellum; BS, brainstem. (d) Double immunofluorescence for $A_{2A}$ receptor ($A_{2A}$R, red) and MAP2 (green) in the striatum showing a dense immunoreactivity for $A_{2A}$ receptor on the surface of some MAP2-labeled somata (#) and dendrites (arrows); the remaining MAP2-labeled somata (*) and dendrites (arrowheads) did not express $A_{2A}$ receptors. (e,f) Triple immunofluorescence for $A_{2A}$ receptor ($A_{2A}$R, red), MAP2 (blue), and enkephalin (Enk, green) or substance P (SP, green) in the striatum showing a selective expression of $A_{2A}$ receptors in Enk-positive medium spiny neurons (#). $A_{2A}$ receptor is not detected in SP-positive medium spiny neurons (*). (g) Triple immunofluorescence for $A_{2A}$R ($A_{2A}$R, red), $D_1$ receptor ($D_1$R, blue), and $D_2$ receptor ($D_2$R, green) in the striatum showing a preferential distribution of $A_{2A}$ in $D_2$ receptor–positive medium spiny neurons (#), but not in $D_1$ receptor–positive medium spiny neurons (*). Scale bars: (b,c) 1 mm; (d–g), 10 µm.

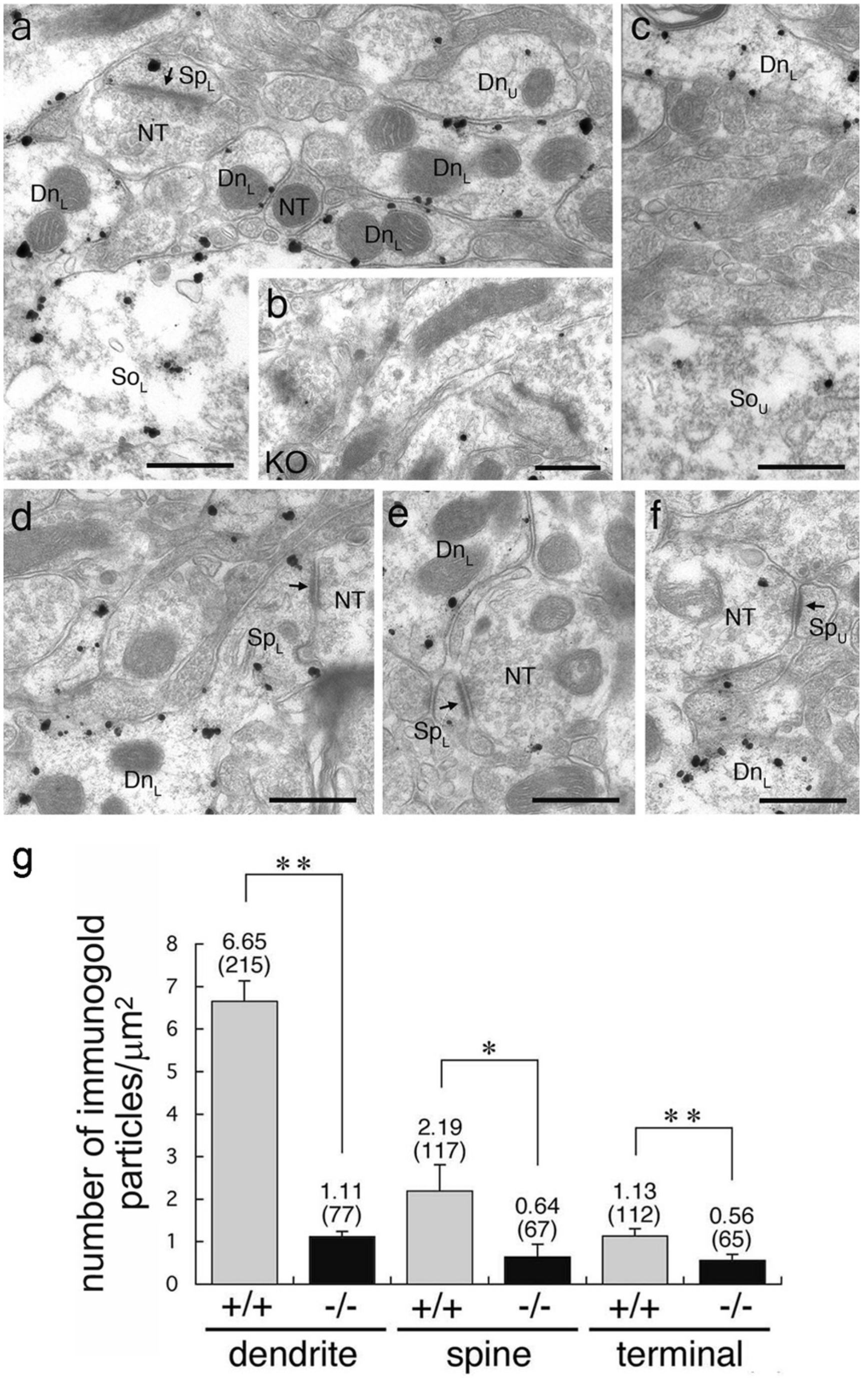

**FIGURE 2.** Electron micrographs of mouse striatum with $A_{2A}$ receptors labeled with silver-enhanced immunogold. (a,c,d) Predominant labeling in some somata (So), dendrites (Dn), and spines (Sp). Strongly labeled elements are indicated by $So_L$, $Dn_L$, and $Sp_L$, while unlabeled or weekly labeled elements are indicated by $So_U$, $Dn_U$, and $Sp_U$. (b) Negative control showing virtual lack of immunogold labeling for $A_{2A}$ receptors in the striatum of $A_{2A}$ receptor knockout mice. (e,f) Less-predominant labeling in nerve terminals (NT) from asymmetric synapses (arrows) with unlabeled spines (f) and more rarely with labeled spines (e). (g) Quantitative analysis of immunolabeling density for $A_{2A}$ receptors in the striatum of wild-type (+/+) and $A_{2A}$ receptor knockout mice (–/–); labeling of terminals is only counted for those forming asymmetric synapses with spines; results are indicated as mean ± SEM; * and **: $p < 0.05$ and $p < 0.01$, respectively. Scale bars: 500 nm.

explains the relatively higher proportion of $A_{2A}$ receptor–immunoreactive synaptic contacts found in the previous study (12% vs. less than 5% in the present study). In any case, the present analysis demonstrated a significantly higher labeling of $A_{2A}$ receptors within the three elements in wild-type vs. $A_{2A}$ receptor knockout mice (Fig. 2g).

With double labeling of $A_{2A}$ receptors with immunogold, and $D_1$ or $D_2$ receptors with immunoperoxidase, we confirmed the strong $A_{2A}$-$D_2$ receptor colocalization and the absence of $A_{2A}$-$D_1$ receptor colocalization at the ultrastructural level (Fig. 3). The diffuse $D_2$ and $D_1$ receptor immunoreactivity was mostly found in dendrites and spines (Fig. 3), and occasionally in some nerve terminals (data not shown). $A_{2A}$ receptor labeling was almost always observed in $D_2$ receptor–positive or $D_1$ receptor–negative profiles and, very rarely, in $D_2$ receptor–negative or $D_1$ receptor–positive profiles (Fig. 3).

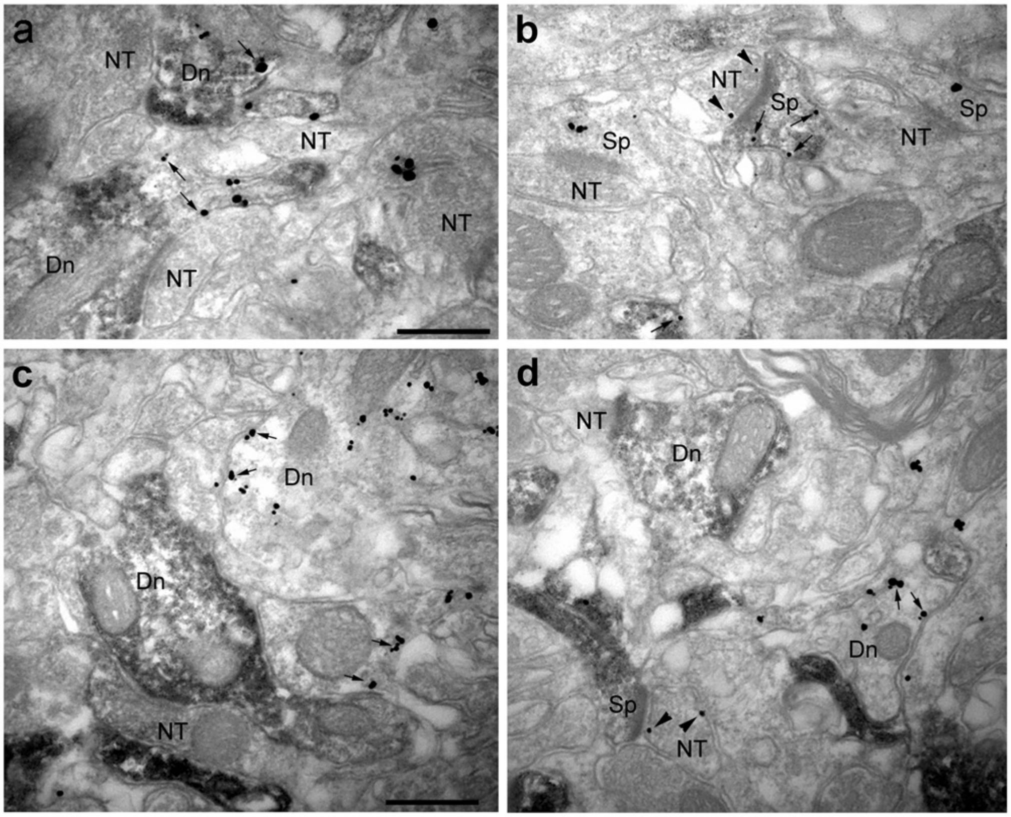

**FIGURE 3.** Electron micrographs of mouse striatum with double labeling of $A_{2A}$ receptors with immunogold, and $D_1$ or $D_2$ receptors with immunoperoxidase. (a,b) Evidence for colocalization of $A_{2A}$ receptors (arrows) and $D_2$ receptors (diffuse dark staining) in dendrites (Dn) and spines (Sp). In (b) there is an example of the uncommon $A_{2A}$ receptor–labeled nerve terminal (NT; arrowheads) making asymmetric synaptic contact with a double-labeled spine. (c,d) Evidence for the lack of colocalization of $A_{2A}$ receptors (arrows) and $D_1$ receptors (diffuse dark staining) in dendrites and spines. In (d) there is an example of an $A_{2A}$ receptor–labeled nerve terminal (arrowheads) making an asymmetric synaptic contact with a $D_1$ receptor–labeled spine. Scale bars: 200 nm.

The previously published analysis already indicated that synaptic contacts between pre- and postsynaptic $A_{2A}$ receptor–labeled elements were rarely observed[21], which, although not commented at that time, was compatible with a presynaptic segregation of $A_{2A}$ receptors, with a preferential expression of $A_{2A}$ receptors in the glutamatergic terminals making synaptic contact with the $D_1$ receptor– vs. the $D_2$ (and $A_{2A}$) receptor–containing MSNs. In the present analysis, some $A_{2A}$ receptor–positive terminals making synaptic contact with $D_1$ receptor– and, less often, $D_2$ receptor–immunoreactive spines could be identified (Fig. 3). The reduced sensitivity imposed by the double-labeling technique made it difficult to quantify the consequently lower density of positive $A_{2A}$ receptor–immunoreactive terminals, with only about 1% of the axon terminals being labeled with immunogold (close to background levels). Nevertheless, we could still observe a higher number of $A_{2A}$ receptor terminals making asymmetric contact with profiles expressing $D_1$ than with those expressing $D_2$ receptors. Analyzing asymmetric synapses with presynaptic $A_{2A}$ immunoreactivity in different double-labeled preparations, 20 out of 25 (80%) synapses showed postsynaptic $D_1$ receptor immunoreactivity, while only five out of 25 (20%) synapses were $D_2$ receptor positive.

We also performed double-labeling experiments in rat striatal sections with $A_{2A}$ receptor (immunoperoxidase) and vGluT1 (immunogold), which represent glutamatergic terminals of cortical origin[33] and found examples of colocalization of both markers in nerve terminals that make asymmetric synapses on $A_{2A}$ receptor immunonegative, but not immunopositive, dendrites (Fig. 4).

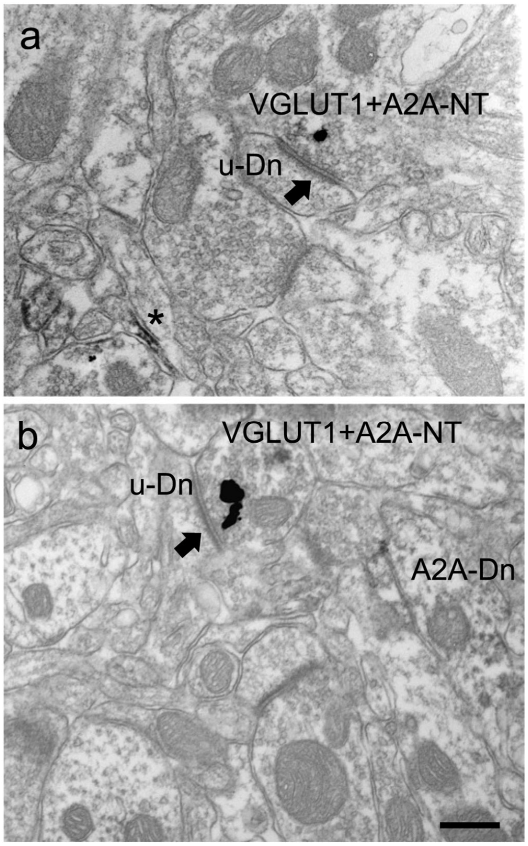

**FIGURE 4.** Electron micrographs of rat striatum with double labeling of $A_{2A}$ receptors with immunoperoxidase and vGluT1 with immunogold. (a,b) Evidence for the existence of nerve terminals (NT) dually labeled for vGluT1 and adenosine $A_{2A}$ receptor (VGLUT1 + A2A-NT) that make asymmetric contact (arrows) on unlabeled dendritic spines (u-Dn). Glutamatergic (vGluT1-containing) nerve terminals are labeled with silver-intensified immunogold and $A_{2A}$ receptor with immunoperoxidase. *: $A_{2A}$ receptor–immunoreactive astrocytic process; $A_{2A}$-Dn: $A_{2A}$-immunoreactive dendrite. Scale = 0.4 μm.

### Immunocytochemical Analysis of $A_{2A}$ Receptors in Striatal Synaptosomes and Nerve Terminals

To provide a quantitative direct demonstration of the preferential localization of presynaptic $A_{2A}$ receptors in corticostriatal glutamatergic terminals facing the direct pathway, we used the simplest preparation to study synaptic events, i.e., synaptosomes[34]. Thus, we carried out an immunocytochemical analysis of $A_{2A}$ receptors in purified nerve terminals (essentially devoid of postsynaptic elements) and in synaptosomes (including both pre- and postsynaptic components) (see Table 1 for summary of results). We confirmed that none of the secondary antibodies produced any signal in preparations to which the addition of the corresponding primary antibody was omitted. Most importantly, we confirmed that the individual signals in double-labeled fields were not enhanced over the signals under single-labeling conditions. Over 70% of the purified nerve terminals (74 ± 6%) or of the synaptosomes (72 ± 2%) visualized were immunopositive for SNAP-25 (n = 4), a SNARE-like protein localized at the plasma membrane of nerve terminals, indicating that both preparations are mostly constituted by presynaptic elements. Less than 2% of elements labeled with synaptophysin (a vesicular protein, widely used as a general synaptic marker[35]) displayed immunoreactivity for the astrocytic marker GFAP (0.75 ± 0.25% for purified nerve terminals and 1.8 ± 0.8% for synaptosomes, n = 4). In accordance with a minor contamination of postsynaptic material in purified nerve terminals, it was found that 8 ± 1% (n = 4) of synaptophysin-immunopositive purified nerve terminals were also immunopositive for PSD95. Also in accordance with the expected presence of postsynaptic material in synaptosomal preparations, it was found that 26 ± 2% (n = 4) of synaptophysin-immunopositive synaptosomes were also immunopositive for PSD95. We confirmed that 27 ± 3% (n = 5) of the purified nerve terminals immunopositive for synaptophysin were immunopositive for vGluT1 and 15 ± 2% (n = 5) were immunopositive for vGluT2, which are markers of striatal glutamatergic synapses, particularly of cortical and thalamic origin, respectively[33]. Likewise, 29 ± 3% (n = 4) of synaptophysin-immunopositive synaptosomes were also immunopositive for vGluT1. This finding that the percentage of putative glutamatergic elements is similar in both synaptosomes and in purified nerve terminals provides further confidence that these two preparations can be compared to evaluate the pre- vs. postsynaptic localization of particular proteins in glutamatergic synapses.

**TABLE 1**
**Immunolabeling of Pre- and Postsynaptic Proteins in Synaptosomal (Mixed Pre- and Postsynaptic Components) and Purified Nerve Terminal Preparations (Enriched Presynaptic Components)**

| Proteins | Synaptosomes | Nerve Terminals |
|---|---|---|
| Snap-25 | 72 ± 2% | 74 ± 6% |
| Synaptophysin + GFAP | 1.8 ± 0.8% | 0.75 ± 0.25% |
| Synaptophysin + PSD95 | 26 ± 2% | 8 ± 1%[a] |
| Synaptophysin + vGluT1 | 29 ± 3% | 27 ± 3% |
| Synaptophysin + vGluT2 | — | 15 ± 2% |
| $A_{2A}$ receptor + vGluT1 | 46 ± 1% | 49 ± 6% |
| $A_{2A}$ receptor + vGluT2 | — | 3.3 ± 0.9%[b] |
| $D_1$ receptor + vGluT1 | 64 ± 5% | 28 ± 8%[a] |
| $D_1$ + $A_{2A}$ + vGluT1 | 48 ± 4% | 21 ± 6%[a] |

[a]Significantly different compared to synaptosomes ($p < 0.01$ in all cases); [b]significantly different compared to A2A receptor + vGluT1; —: not analyzed.

It was found that vGluT1-positive synaptosomes had a significantly higher expression of $D_1$ receptors than vGluT1-positive purified nerve terminals (64 ± 5% vs. 28 ± 8%, respectively; $p < 0.01$; n = 4). These results confirm that $D_1$ receptors are more abundant postsynaptically (in synaptosomes) than presynaptically (in purified nerve terminals) in these glutamatergic synapses, thus allowing reliable allocation of proteins in glutamatergic synapses impinging on direct pathway MSNs. This agrees with the well-known predominant postsynaptic localization of $D_1$ receptors in the striatum and also confirms the existence of a low, albeit significant, expression of $D_1$ receptors in glutamatergic terminals[36,37]. That was not the case for $D_2$ receptors, which showed a similar expression in both preparations (data not shown). Importantly, the percentage of vGluT1-positive nerve terminals that were also positive for $A_{2A}$ receptors was as high as 49 ± 6% (n = 4), while a significantly much lower proportion of vGluT2-positive nerve terminals were also positive for $A_{2A}$ receptors (3.3 ± 0.9%; $p < 0.001$; n = 4). This result confirms the predominant localization of presynaptic striatal $A_{2A}$ receptors in cortical vs. thalamic glutamatergic terminals. Triple labeling revealed that only 21 ± 6% (n = 4) of vGluT1-positive purified nerve terminals were also simultaneously endowed with immunoreactivity for $A_{2A}$ and $D_1$ receptors, whereas a significantly higher number of vGluT1-positive synaptosomes, 48 ± 4% ($p < 0.01$; n = 4), were simultaneously endowed with $A_{2A}$ and $D_1$ receptor immunoreactivity (Fig. 5). Since $D_1$ receptors are mostly postsynaptic and not colocalized with $A_{2A}$ receptors, the higher proportion of tripled-labeled synaptosomes can only be explained by a preferential expression of $A_{2A}$ receptors in cortical glutamatergic terminals impinging on the direct pathway.

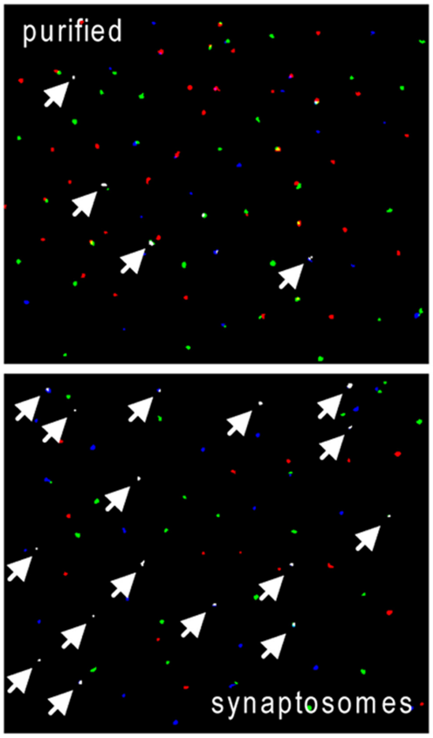

**FIGURE 5.** $A_{2A}$ receptors in striatal synaptosomes and nerve terminals from rat striatum. Representative triple-labeling immunocytochemical colocalization of adenosine $A_{2A}$ receptor (blue), $D_1$ receptor (red), and $vGluT_1$ (green) in synaptosomes and purified nerve terminals; in white, triple colocalization (arrows). There was a significantly higher triple expression of vGluT1 and $A_{2A}$ and $D_1$ receptors in synaptosomes compared to purified nerve terminal preparations (see Table 1 and text).

### Electrophysiological Analysis of the Role of Striatal $A_{2A}$ Receptors on Excitatory Transmission to the Direct and Indirect Pathways

The next step was to find a functional demonstration of the preferential $A_{2A}$ receptor–mediated presynaptic control of glutamatergic neurotransmission in the direct pathway. We prepared brain slices containing the dorsal striatum from $M_4$-GFP and $D_2$-GFP BAC transgenic mice, which label direct- and indirect-pathway MSNs, respectively[38]. Bath application of the $A_{2A}$ receptor agonist CGS21680 (1 µ*M*) significantly increased the amplitude of EPSCs measured in direct-pathway MSNs (123 ± 8% of baseline, n = 7, $p < 0.05$; Fig. 6a,b), whereas it had no effect on EPSCs measured from indirect-pathway MSNs (98 ± 7% of baseline, n = 5, $p > 0.05$; Fig. 6f). Previous studies suggest the existence of an $A_{2A}$ receptor–mediated presynaptic inhibitory control of intrastriatal GABAergic synaptic transmission[39], which could increase glutamatergic neurotransmission by an indirect mechanism, releasing a $GABA_B$ receptor–mediated inhibition of glutamate release[40]. Nevertheless, under our experimental conditions, CGS21680 failed to modulate inhibitory postsynaptic currents (IPSCs) measured in direct-pathway MSNs (95 ± 10% of baseline, n = 5, $p > 0.05$; Fig 6d). Since ATP is probably coreleased with some striatal neurotransmitters and quickly converted to adenosine (see Discussion), we next tested whether antagonizing $A_{2A}$ receptors could modulate EPSCs. Application of the selective $A_{2A}$ receptor antagonist SCH442416 (1 µ*M*) significantly decreased EPSC amplitudes in direct-pathway MSNs (85 ± 5% of baseline, n = 6, $p < 0.05$; Fig. 6c), but had no effect on indirect-pathway MSNs (102 ± 20% of baseline, n = 5, $p > 0.05$; Fig. 6d). Furthermore, there was no change in EPSC amplitude if CGS21680 (1 µ*M*) was applied in the presence of SCH442416 (1 µ*M*; 102 ± 7% of baseline, n = 5; data not shown). To determine whether the observed changes were presynaptic, we performed mean-variance analysis[25,26]. Postsynaptic changes (q) should yield no change in the CV ratio (i.e., should remain at 1), whereas presynaptic changes (N or p) should lie along or above the diagonal line (for increases in synaptic strength), or along or below the diagonal line (for decreases in synaptic strength). Following application of CGS21680, the CV ratio was significantly greater than 1 (1.49 ± 0.2, n = 7, $p < 0.05$; Fig. 6e), indicating a presynaptic site of modulation.

### Analysis of the Role of Striatal $A_{2A}$ Receptors on the Control of Glutamate Release Induced by Cortical Stimulation

Basal extracellular levels of glutamate and dopamine in the lateral striatum were 2.7 ± 0.4 µ*M* and 4.4 ± 0.2 n*M*, respectively (n = 22). The extracellular levels of both glutamate and dopamine significantly increased (50 and 20%) in the lateral striatum after 20 min of electrical stimulation in the orofacial area of the motor cortex (Fig. 7a,b). The levels returned to the baseline after the offset of the stimulation. This effect was constrained to the projecting area of the cortex that was being stimulated. Thus, there was no significant increase of glutamate or dopamine in the adjacent medial striatum (Fig. 7a,b). Perfusion with MSX-3 (1 µ*M*) did not modify basal levels of glutamate or dopamine, and perfusion with the $D_1$ receptor antagonist SCH23390 (1 µ*M*) did not modify glutamate levels, but significantly increased dopamine levels (by 20%), which remained constant until application of cortical electrical stimulation. This effect of SCH23390 at least confirms that the concentration used was effective at blocking dopamine receptors. Fig. 7 shows normalized values, expressed as percentage of the mean of the three values before cortical electrical stimulation. Two-way repeated measures ANOVA showed a significant treatment effect ($p < 0.001$), due to a significant difference between the MSX-3–treated group vs. the other three groups. There was also a significant interaction between the treatment and time effects ($p < 0.01$). Cortical stimulation induced an increase in the values of glutamate and dopamine in the lateral striatum (posthoc Newman-Keuls test: $p < 0.01$ and $p < 0.05$ vs. values previous to stimulation), and this effect was significantly reduced with perfusion with MSX-3 (1 µ*M*), but not with the $D_1$ receptor antagonist SCH23390 (1 µ*M*) (Fig. 7a,b).

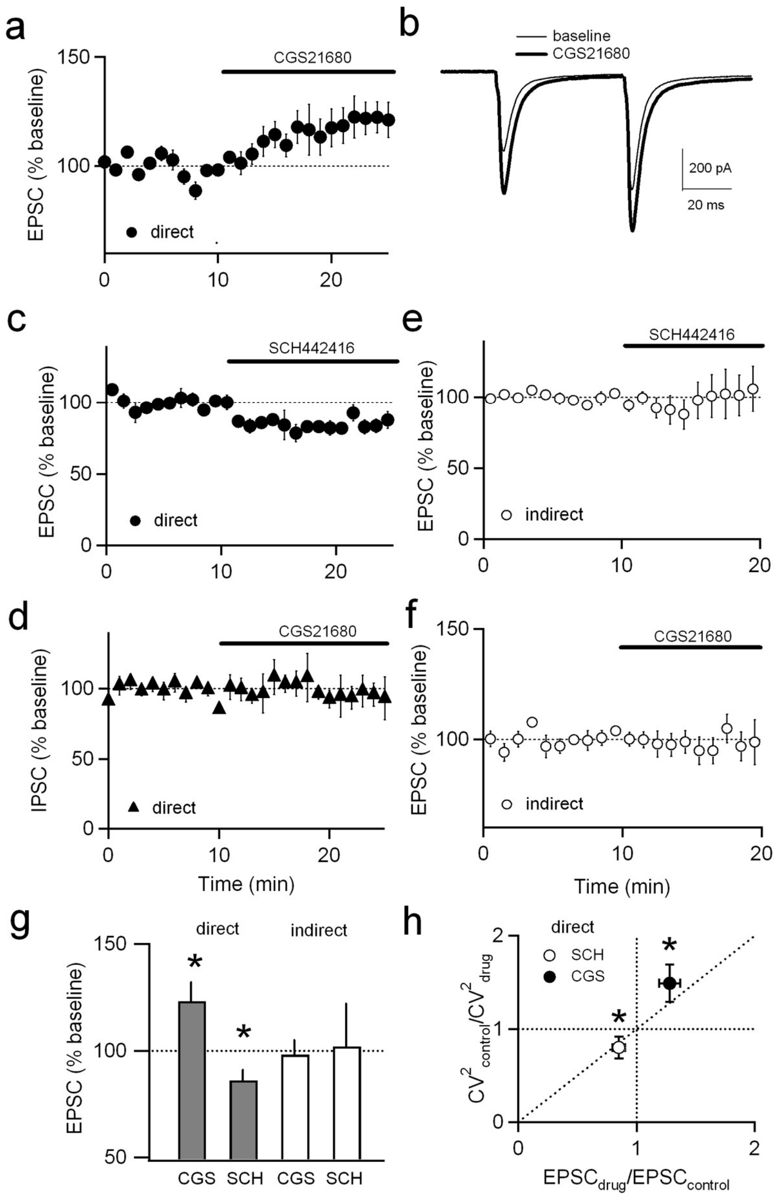

**FIGURE 6.** $A_{2A}$ receptor activation enhances glutamatergic transmission at direct-pathway striatal GABAergic efferent neurons. (a) Normalized mean EPSC amplitudes recorded from direct-pathway MSNs were significantly enhanced by CGS21680 (1 μ*M*) (n = 7, $p < 0.05$). (b) Average traces recorded at baseline (thin line) and after CGS21680 bath application (thick line). (c) The $A_{2A}$ receptor antagonist SCH442416 (1 μ*M*) significantly decreased EPSC amplitude (n = 6, $p < 0.05$) at direct-pathway MSNs. (d) CGS21680 did not alter IPSC amplitudes (n = 5, $p > 0.05$). (e) SCH442416 had no effect on normalized mean indirect-pathway EPSC amplitude (n = 5, $p > 0.05$). (f) CGS21680 had no effect on normalized mean indirect-pathway EPSC amplitude (n = 5, $p > 0.05$). (g) Summary of EPSC modulation by CGS21680 (CGS) and SCH442416 (SCH) at 20–25 min (direct pathway) or 15–20 min (indirect pathway) after drug application. (h) Mean-variance analysis of direct-pathway EPSCs following drug application (20–25 min). Asterisks denote $p < 0.05$. Results are expressed as mean ± SEM.

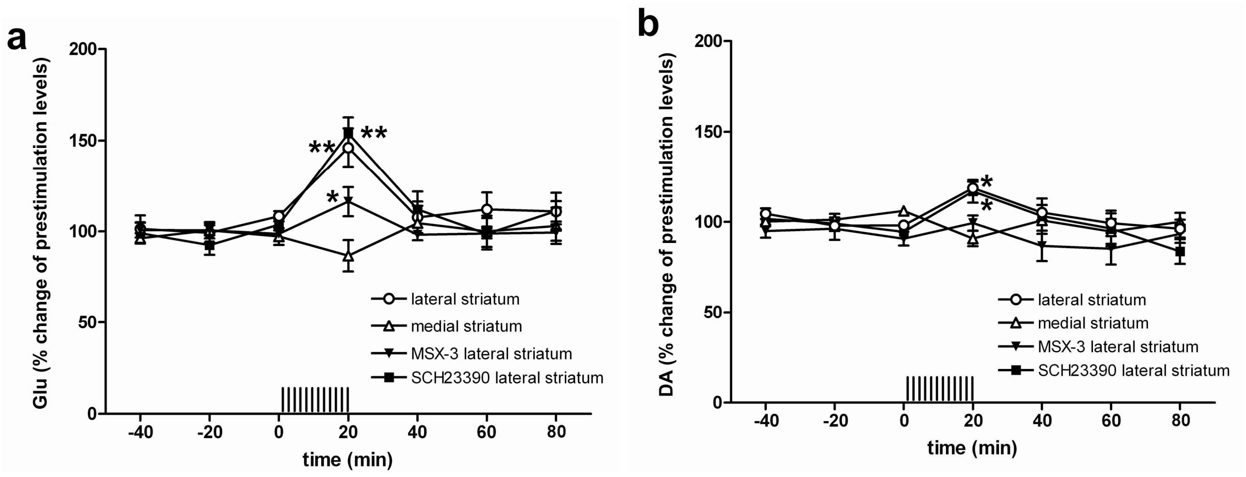

**FIGURE 7.** $A_{2A}$ receptor antagonist–induced blockade of striatal glutamate release induced by cortical electrical stimulation. Extracellular levels of glutamate (a) and dopamine (b) in the striatum after cortical electrical stimulation. Cortical stimulation in the orofacial area of the motor cortex produced a significant increase in the extracellular concentrations of glutamate (Glu) and dopamine (DA) in the lateral, but not the medial, striatum. Perfusion of the $A_{2A}$ receptor antagonist MSX-3, but not the $D_1$ receptor antagonist SCH23390, significantly counteracted the increase in glutamate and dopamine extracellular levels in the lateral striatum induced by cortical electrical stimulation (repeated measures ANOVA: $p < 0.05$). Results represent means ± SEM of percentage of the mean of the three values before the stimulation; n = 5–10 per group. Time '0' represents the values of the samples previous to the stimulation. The train of vertical lines represents the period of cortical stimulation. *, **: significantly different ($p < 0.05$ and $p < 0.01$, respectively; Newman-Keuls posthoc tests) compared to value of the last sample before the stimulation.

### Analysis of the Role of Striatal $A_{2A}$ Receptors on the Motor Output Induced by Cortical Electrical Stimulation

The parameters of cortical electrical stimulation were set to produce the minimal visible somatic jaw movements. In addition to the blockade of glutamate and dopamine release, the striatal perfusion of MSX-3 blocked jaw movements induced by cortical stimulation in nine out of 10 animals. To perform a quantitative measurement of the effects induced by $A_{2A}$ receptor blockade on the motor output induced by cortical electrical stimulation, we established a PCC as a method of analysis of correlation between the power of the input signals (current pulses) and the power of output signals obtained from the measurement of EMG activity from the jaw muscles (see Methods and Fig. 8). PCC was found to be a very stable and reproducible variable among different animals (see, for instance, the very similar PCC values of the two different control groups in Fig. 8c,d). Systemic administration of either the $A_{2A}$ receptor antagonist MSX-3 or the $D_1$ receptor antagonist SCH23390 produced a significant dose-dependent decrease in PCC (Fig 8c,b) at behaviorally relevant doses[41,42].

### Analysis of the Role of Striatal $A_{2A}$ Receptors on the Striatal Protein Phosphorylation Induced by Cortical Electrical Stimulation

As previously reported[28], cortical stimulation produced a significant increase in ERK1/2 phosphorylation and in PKA-dependent phosphorylation of the $GluR_1$ subunit of the AMPA receptor (Fig. 9). In addition, systemic administration of MSX-3 (3 mg/kg, i.p.) significantly counteracted both ERK1/2 and $GluR_1$ phosphorylation, as previously found[28] (Fig. 9). On the other hand, the $D_1$ receptor antagonist SCH23390 (0.3 mg/kg, i.p.) counteracted $GluR_1$, but not ERK1/2, phosphorylation (Fig. 9).

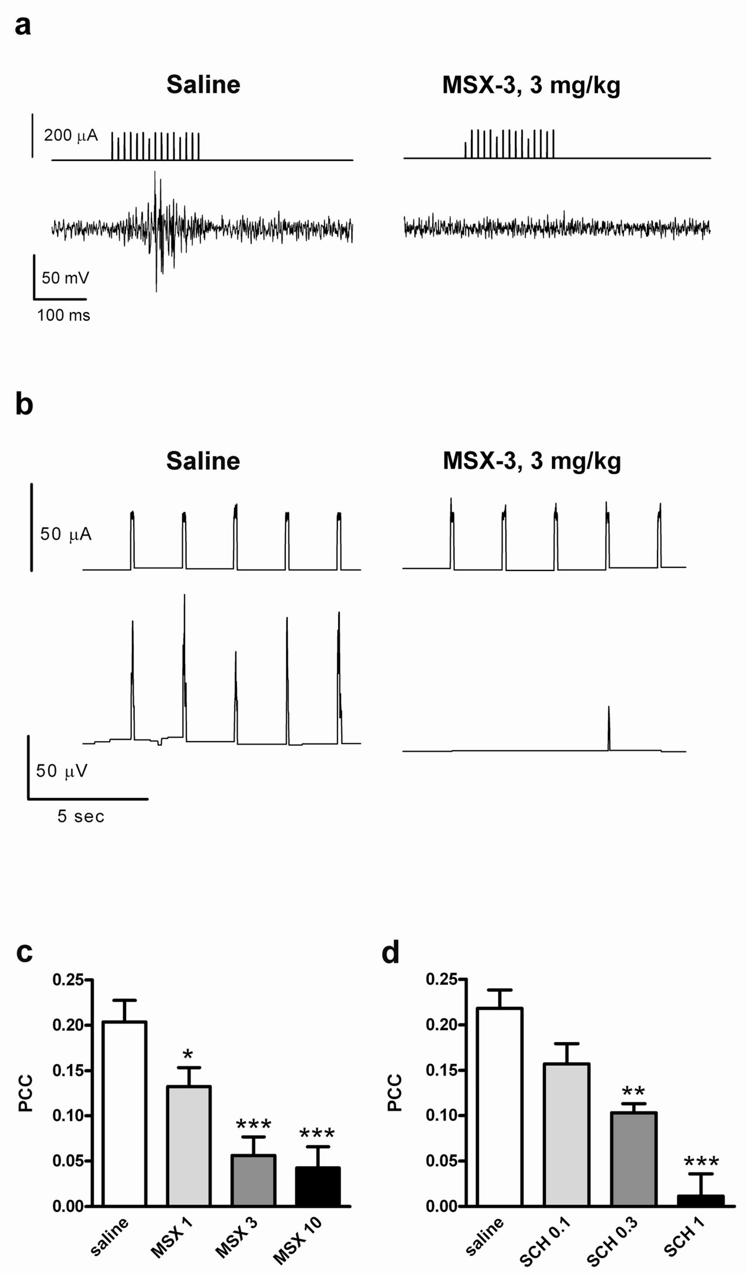

**FIGURE 8.** $A_{2A}$ receptor antagonist–induced blockade of the motor output induced by cortical electrical stimulation. (a) Representative recordings of the input signal (current pulses) delivered from the stimulator in the orofacial area of the motor cortex (upper traces) and the EMG output signal obtained from the jaw muscles (lower traces), after the administration of either saline (left traces) or the $A_{2A}$ receptor antagonist MSX-3 (3 mg/kg, i.p.; right traces). (b) Representative input stimulator signal power (time constant 0.01 sec; upper traces) and output EMG signal power (time constant 0.01 sec; lower traces) after the administration of either saline (left traces) or MSX-3 (right traces). (c,d) Dose-dependent decrease in the PCC of the power of input and output signals induced by the administration of MSX-3 (c) and the $D_1$ receptor antagonist SCH23390 (d). Results represent means ± SEM (n = 6–8 per group); *, **, ***: significantly different compared to saline ($p < 0.05$, $p < 0.01$, and $p < 0.001$, respectively).

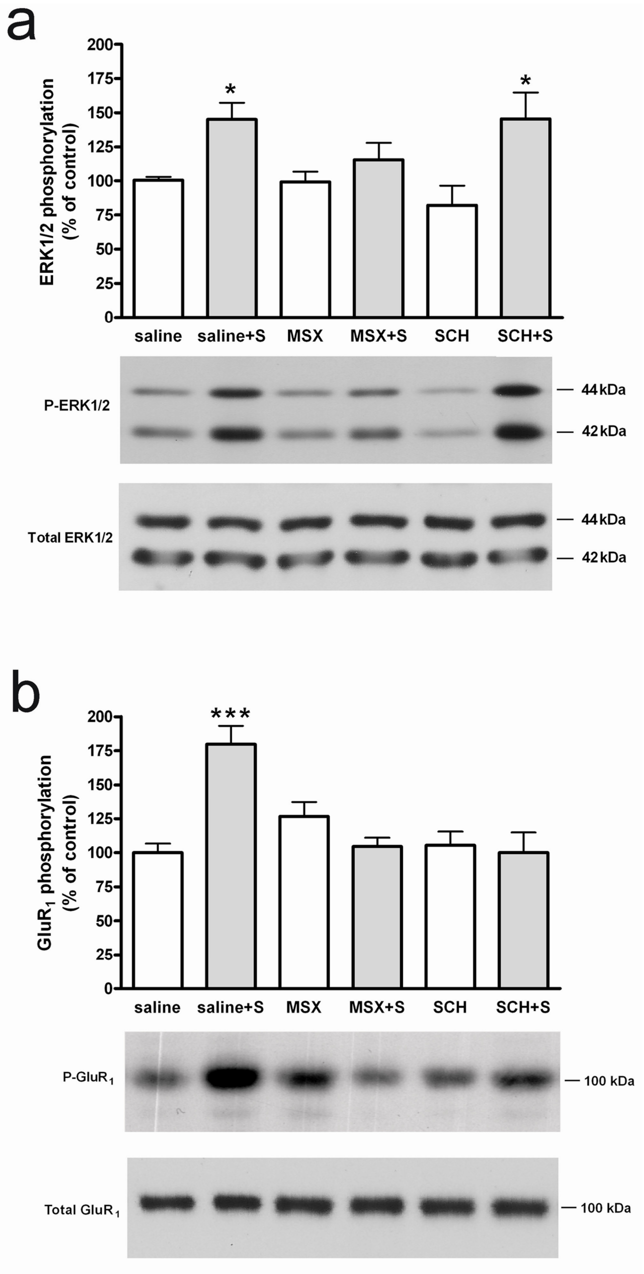

**FIGURE 9.** $A_{2A}$ receptor antagonist–induced blockade of the striatal protein phosphorylation induced by cortical electrical stimulation. (a) The $A_{2A}$ receptor antagonist MSX-3 (3 mg/kg), but not the $D_1$ receptor antagonist SCH23390 (0.3 mg/kg), counteracted striatal ERK1/2 phosphorylation induced by cortical stimulation. (b) Both MSX-3 and SCH23390 counteracted striatal PKA-dependent phosphorylation of $GluR_1$ subunit of AMPA receptor. Results are shown in means ± SEM (n = 6–13 per group) and representative Western blots. *, ***: significantly different compared to saline-treated group ($p < 0.05$ and $p < 0.001$, respectively). "S" refers to cortical stimulation.

### DISCUSSION

Prior *in situ* hybridization experiments provided indirect evidence for a preferential localization of the $A_{2A}$ receptor protein in the $D_2$ receptor–containing indirect-pathway MSN[30,31,32,43]. We developed a new $A_{2A}$ receptor antibody with a high degree of specificity and very effective for immunohistochemical electron microscopy analysis, and for the first time we obtained direct evidence for the dense localization of $A_{2A}$ receptor protein in indirect-pathway MSNs and their absence in $D_1$ receptor–containing direct-pathway MSNs. As in previous ultrastructural analyses[21,44], electron microscopy showed that $A_{2A}$ receptors were preferentially localized in dendrites and dendritic spines, and less prevalent in axon terminals, and that the large majority of synaptic contacts containing $A_{2A}$ receptor immunoreactivity were asymmetric synapses. In striatal nerve terminal preparations, $A_{2A}$ receptors colocalized with vGluT1, but not with vGluT2, indicating that presynaptic $A_{2A}$ receptors are localized in corticostriatal, but not thalamostriatal, terminals[33]. The $A_{2A}$ receptor–vGluT1 colocalization was also observed in electron microscopy experiments. By utilizing electron microscopy experiments with double immunolabeling of $A_{2A}$ and $D_1$ or $D_2$ receptors, we could identify asymmetric synapses with presynaptic $A_{2A}$ and postsynaptic $D_1$ receptors more often than asymmetric synapses with presynaptic $A_{2A}$ and postsynaptic $D_2$ receptors. The predominant presynaptic localization of $A_{2A}$ receptors in the corticostriatal glutamatergic terminals targeting the direct-pathway MSNs was confirmed in triple immunocytochemical studies labeling with vGluT1, $A_{2A}$ receptors, and $D_1$ receptors in striatal synaptosomes and purified nerve terminal preparations.

With patch-clamp experiments in identified direct- and indirect-pathway MSN neurons, we could also provide a functional demonstration of the segregation of striatal presynaptic $A_{2A}$ receptors in the two striatal pathways. An $A_{2A}$ receptor agonist and an $A_{2A}$ receptor antagonist significantly increased and decreased, respectively, the amplitude of EPSCs induced by the intrastriatal stimulation of glutamatergic afferents measured in identified direct-, but not indirect-, pathway MSNs. Mean-variance analysis indicated a presynaptic locus for $A_{2A}$ receptor–mediated modulation. Together with the immunohistochemical and immunocytochemical data, these results demonstrate that striatal $A_{2A}$ receptors selectively regulate excitatory transmission at direct-pathway synapses through presynaptic modulation of glutamate release.

Further functional confirmation of the presynaptic $A_{2A}$ receptor–mediated modulation of corticostriatal glutamatergic neurotransmission came from *in vivo* microdialysis experiments. The intrastriatal perfusion of an $A_{2A}$ receptor antagonist significantly counteracted striatal glutamate and dopamine release induced by cortical stimulation. The apparently stronger $A_{2A}$ receptor–mediated modulation of striatal glutamatergic neurotransmission shown in the microdialysis compared to the slice experiments is most probably due to the fact that in the first case, there is a selective stimulation of glutamate release from corticostriatal terminals, while in the slice experiments, there is also stimulation of the thalamostriatal terminals, which, according to immunocytochemical analysis, do not show a significant presence of $A_{2A}$ receptors. Considering also the results from the morphological experiments, the results obtained from patch-clamp and *in vivo* microdialysis experiments indicate that cortical stimulation produces a preferential release of striatal glutamate from the glutamatergic terminals that innervate the direct-pathway MSNs and that this release is under the control of presynaptic $A_{2A}$ receptors. The ability of the local perfusion of the $A_{2A}$ receptor antagonist to counteract dopamine release (in addition to glutamate release) induced by cortical stimulation suggests that striatal dopamine release induced by cortical stimulation is mostly dependent on glutamate release, since there is no evidence for the presence of functional $A_{2A}$ receptors in dopaminergic terminals. This supports previous results obtained with different experimental approaches that suggested the local ability of striatal glutamate to modulate dopamine release by acting on ionotropic glutamate receptors localized in dopaminergic terminals[45,46,47].

A striking unexpected finding was that intrastriatal perfusion of an $A_{2A}$ receptor antagonist through the microdialysis probe not only counteracted striatal glutamate release, but also jaw movements induced by cortical electrical stimulation. By combining cortical electrical stimulation and recording of EMG

activity of the mastication muscles, we established a PCC as an objective *in vivo* measure of corticostriatal neurotransmission. PCC was shown to be significantly and dose dependently decreased by the systemic administration of an $A_{2A}$ receptor antagonist. According to the widely accepted functional basal circuitry model (see Introduction), blockade of postsynaptic $A_{2A}$ receptors localized in the GABAergic striatopallidal neurons of the indirect pathway (by potentiating $D_2$ receptor–mediated effects in the $D_2$ receptor–containing MSNs by means of $A_{2A}$-$D_2$ receptor interactions[7,9,12,13,14]) should potentiate spontaneous or psychostimulant-induced motor activation. On the other hand, according to the same model, blockade of presynaptic $A_{2A}$ receptors localized in the corticostriatal glutamatergic terminals that make synaptic contact with the direct pathway should decrease motor activity. The well-known preferential locomotor activating effects of systemically administered $A_{2A}$ receptor antagonists could be explained by a stronger influence of a tonic adenosine and $A_{2A}$ receptor–mediated modulation of the indirect pathway vs. the direct pathway under basal conditions. The recent results by Shen et al.[48] about the differential effects of $A_{2A}$ receptor antagonists on psychostimulant-induced locomotor activation in wild-type vs. striatal postsynaptic $A_{2A}$ receptor–specific knockout mice (potentiating vs. counteracting effects, respectively) support this hypothesis. Activation of presynaptic $A_{2A}$ receptors seems to be highly dependent on adenosine released upon strong corticostriatal glutamatergic input[12,15,16]. Therefore, one would expect $A_{2A}$ receptor antagonists to counteract motor activation induced by strong corticostriatal glutamatergic neurotransmission, such as orofacial movements induced by cortical electrical stimulation. According to this interpretation, we also found that the $D_1$ receptor antagonist SCH23390, which is known to induce motor depression by blocking postsynaptic $D_1$ receptors localized in direct-pathway MSNs, also produces a significant decrease in PCC.

We have previously reported that $A_{2A}$ receptor antagonists block striatal ERK1/2 phosphorylation and PKA-dependent phosphorylation of the $GluR_1$ subunit of the AMPA receptor induced by cortical electrical stimulation[28]. It has been demonstrated that cortical stimulation induces ERK1/2 phosphorylation in the indirect-, but not in the direct-, pathway MSNs[49]. Since our results indicate that there are no presynaptic $A_{2A}$ receptors regulating glutamatergic neurotransmission to the indirect pathway, $A_{2A}$ receptor antagonist–induced blockade of ERK1/2 phosphorylation induced by cortical stimulation must depend on blockade of postsynaptic $A_{2A}$ receptors, which are selectively localized in indirect-pathway MSNs. On the other hand, the present results suggest that postsynaptic $A_{2A}$ receptors are not involved in the $A_{2A}$ receptor antagonist–induced blockade of PKA-dependent phosphorylation of AMPA receptors after cortical electrical stimulation. The role of $D_1$ receptors in PKA-dependent phosphorylation of AMPA receptors in direct-pathway MSNs is quite well established[50]. In our experimental setting, a $D_1$ receptor antagonist completely counteracted PKA-dependent phosphorylation of AMPA receptors, which most probably depends on the glutamate-dependent dopamine release induced by cortical electrical stimulation (see above). Therefore, the most probable scenario is that cortical electrical stimulation selectively induces a $D_1$ receptor–mediated, PKA-dependent phosphorylation of AMPA receptors in direct-pathway MSNs and that its counteraction by $A_{2A}$ receptor antagonists depends on the counteraction of glutamate-dependent dopamine release secondary to the blockade of presynaptic $A_{2A}$ receptors.

The present study provides morphological and functional evidence for a key role of presynaptic $A_{2A}$ receptors in the modulation of cortical glutamatergic neurotransmission to the striatal direct efferent pathway. The main sources of extracellular adenosine in the striatum still need to be established, but there is evidence suggesting that an important source is ATP coreleased with glutamate from glia and glutamatergic terminals[9,51]. This ATP would then be converted to adenosine by means of ectonucleotidases[9,51]. Recent studies also suggest that ATP could also be coreleased with dopamine in the striatum[52]. The present results indicate that the predominant expression of pre- and postsynaptic $A_{2A}$ receptors in the glutamatergic synapses of the direct- and indirect-pathway neurons, respectively, allows adenosine to play an elaborated role in striatal function. The results also suggest that while a more selective blockade of postsynaptic striatal $A_{2A}$ receptors could provide useful antiparkinsonian agents[53,54], selective striatal presynaptic $A_{2A}$ receptor antagonists could be beneficial in conditions where a decreased cortical neurotransmission to the direct pathway would be desirable, such as Huntington's disease and L-dopa–induced dyskinesias[55].

### ACKNOWLEDGMENTS

The authors would like to thank Mrs. María José Cabañero for the excellent technical assistance and Dr. Robert H. Edwards, UCSF, San Francisco, for kindly providing the vGluT1 receptor antibody. Work supported by the intramural funds of the National Institute on Drug Abuse, CHDI Foundation, the "Consejería de Educación y Ciencia de la Junta de Comunidades de Castilla-La Mancha" (PAI08-0174-6967) to RL, Fundação para a Ciência e Tecnologia (FCT-PTDC/SAU-NEU/81064/2006) to RAC, National Institutes of Health RO1 NS054978.
